# Influence of the Postcuring Process on Dimensional Accuracy and Seating of 3D-Printed Polymeric Fixed Prostheses

**DOI:** 10.1155/2020/2150182

**Published:** 2020-11-13

**Authors:** Jaewon Kim, Du-Hyeong Lee

**Affiliations:** ^1^Department of Periodontics and Endodontics, School of Dental Medicine, University at Buffalo, New York, USA; ^2^Department of Prosthodontics, School of Dentistry, Institute for Translational Research in Dentistry, Kyungpook National University, Daegu 41940, Republic of Korea

## Abstract

The postcuring process is essential for 3-dimensional (3D) printing of photopolymer-based dental prostheses. However, the deformation of prostheses resulting from the postcuring process has not been fully investigated. The purpose of this study was to evaluate the effects of different postcuring methods on the fit and dimensional accuracy of 3D-printed full-arch polymeric fixed prostheses. A study stone model with four prosthetic implant abutments was prepared. A full-arch fixed dental prosthesis was designed, and the design was transferred to dental computer-aided manufacturing (CAM) software in which supports were designed to the surface of the prosthesis design for 3D printing. Using a biocompatible photopolymer and a stereolithography apparatus 3D printer, polymeric prostheses were produced (*N* = 21). In postcuring, the printed prostheses were polymerized in three different ways: the prosthesis alone, the prosthesis with supports, or the prosthesis on a stone model. Geometric accuracy of 3D-printed prostheses, marginal gap, internal gap, and intermolar distance was evaluated using microscopy and digital techniques. Kruskal-Wallis and Mann-Whitney *U* tests with Bonferroni correction were used for the comparison of results among groups (*α* = 0.05). In general, the mean marginal and internal gaps of cured prostheses were the smallest when the printed prostheses were cured with seating on the stone model (*P* < 0.05). With regard to the adaptation accuracy, the presence of supports during the postcuring process did not make a significant difference. Error in the intermolar distance was significantly smaller in the model seating condition than in the other conditions (*P* < 0.001). Seating 3D-printed prosthesis on the stone model reduces adverse deformation in the postcuring process, thereby enabling the fabrication of prostheses with favorable adaptation.

## 1. Introduction

Interim fixed dental prostheses are usually made in clinics with autopolymerizing acrylic resins [1, 2]. This conventional manual methodology is still the mainstream approach in fabricating fixed prosthodontics but is labor-intensive and uncomfortable for patients because the direct fabrication of prostheses is performed inside the patient's mouth, and heating occurs during polymerization. Because of these drawbacks, digital scanning and computer-aided design/computer-aided manufacturing (CAD/CAM) technologies are increasingly being used to fabricate interim polymeric prostheses [3, 4]. The oral anatomic shape is virtually registered using an optical scanner, and the scan data are imported into dedicated dental CAD software, in which the cementation space of the prosthetic crown is set and a final prosthesis is designed [5]. The design is then transferred to CAM software where the 3D image is divided into 2D cross-sectional images and processed to the polymeric prosthesis using additive manufacturing technologies [6]. The final fabrication process is the postcuring treatment of the printed prosthesis [6].

The 3D printing technologies have diversified treatment procedures and have become an alternative to manual and subtractive methods in medical and dental fields [7–10]. There are several different ways to print polymeric prostheses, such as stereolithography apparatus (SLA), digital light processing, fused deposition modeling, and polymer jetting [11, 12]. The SLA printing method uses liquid photopolymer, and objects are built layer-by-layer using site-specific polymerization by an ultraviolet laser [13]. For the production of interim dental prostheses via SLA printing, several photocurable resins are available and approved for long-term intraoral use [6, 14]. Commonly used acrylic resins could be cytotoxic to the human body in the uncured state [15], but the biocompatibility significantly improves after postcuring and cleaning in the 3D-printed objects [6, 16]. The quality of 3D-printed objects significantly varies depending on operational parameters, fabrication workflow, materials, and devices [16, 17]. The contemporary 3D-printed polymers for interim dental applications exhibit low anisotropy and appropriate properties required for the end product [6, 14]. The mechanical and physicochemical properties of photopolymers available on the market are reported to be comparable to those of conventional autopolymerizing acrylic resins [6, 8].

The postcuring process increases the degree of polymerization of the printed object, which affects the final mechanical properties and the amount of the object's residual monomer [6, 14, 16]. However, postcuring can also cause dimensional deformation in the general structure and warping in any thin areas of an object because of the inherent change of the chemical bonds during polymerization [18]. Although the postcuring process is essential for the photopolymer-based 3D printing of dental prostheses, whether or not different postcuring methods affect the geometric accuracy of the prosthesis has not been fully investigated. The purpose of this study was to evaluate the effects of different postcuring conditions on the geometric accuracy of fabrication of full-arch polymeric fixed prostheses that were created using SLA 3D printing. The adaptation of prostheses on abutments and dimensional deformation were assessed by means of a marginal gap, internal gap, and intermolar distance. The null hypothesis was that the differences in the postcuring methods for printed polymeric prostheses would not affect the accuracy of their fabrication.

## 2. Materials and Methods

### 2.1. Fabrication of the Study Model and Full-Arch Polymeric Prostheses

**Figure 1:**
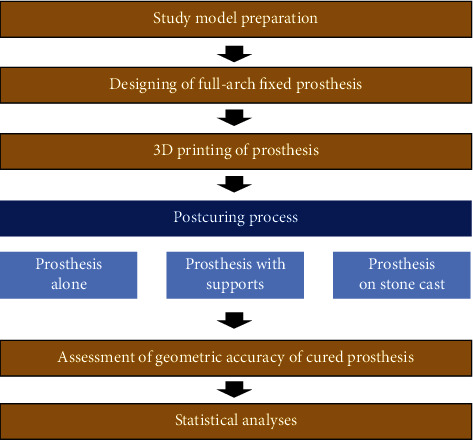
Workflow of this study.

**Figure 2:**
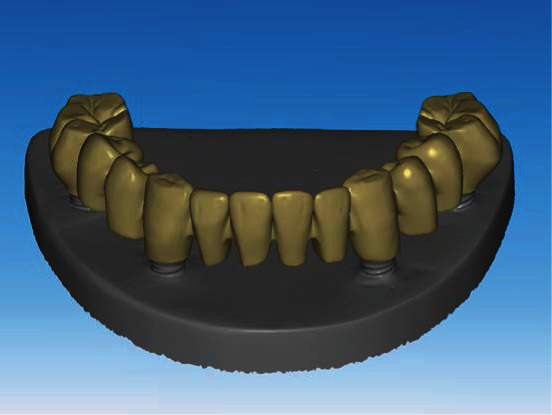
Design of implant-supported full-arch fixed dental prosthesis.

**Figure 3:**
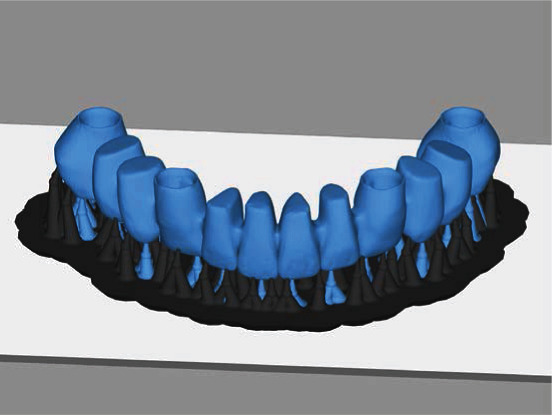
Insertion of support structures into the prosthesis design for the 3D printing procedure.

The overall study procedure is described in Figure 1. The edentulous study stone model was prepared, with four prosthetic implant abutments (FreeForm ST; Osstem, Seoul, Korea) that were connected to implants (USII; Osstem) placed in the canine and second premolar areas. A virtual model was created by digitizing the surface of the stone model using a laboratory-based scanner (IDC S1; Amann Girrbach, Koblach, Austria) and was transferred to a dental design software program (R2CAD; MegaGen, Daegu, Korea), which designed a 12-unit implant-supported fixed dental prosthesis (Figure 2). The design file was transferred to a CAM software program for 3D printing (Raydent Studio; Ray, Hwaseong-si, Korea), in which supports were installed on the occlusal surface of the prosthesis design (Figure 3). Subsequently, interim acrylic prostheses were produced by printing a biocompatible photopolymer (Raydent C&B; Ray) in a SLA 3D printer (Meg-Printer II; MegaGen) with a layering thickness of 50 *μ*m and with a wavelength of 405 nm (Table 1). The printed prostheses were then rinsed thoroughly under running water and spray dried at room temperature according to the manufacturer's instructions.

### 2.2. Postcuring Process of 3D-Printed Prostheses

**Figure 4:**
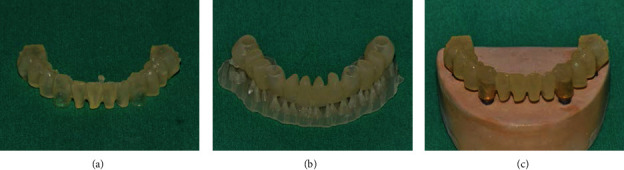
Postcuring methods: (a) prosthesis alone, (b) prosthesis with supports, and (c) prosthesis on the stone model.

In the postcuring procedure, the printed prostheses were polymerized in an ultraviolet curing unit of the 3D printer for 15 minutes with a wavelength of 395 nm and radiation power at 60 mW/cm^2^. Three different methods were used for the postcuring procedure (*n* = 7 in each group; *N* = 21) (Figure 4): prosthesis alone (P group), prosthesis with supports (PS group), and prosthesis on the stone model (PM group). In the P group, supports were removed from the printed prosthesis using a cutter, and postcuring was performed. In the PS group, the prosthesis was cured without removing the supports. In the PM group, supports were removed, and the printed prosthesis was seated on the prosthetic abutments of a stone model, and then, the postcuring was performed. For random sampling, the printed prostheses were allocated to each group consecutively in the order of fabrication. All 3D printing and postcuring processes were performed by a single operator (D.H.L.).

### 2.3. Evaluation of Fabrication Accuracy of Prostheses

**Figure 5:**
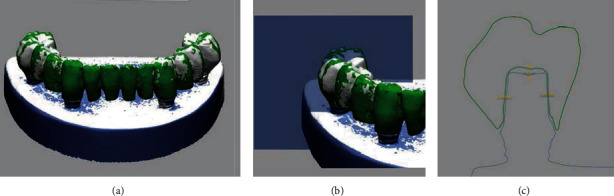
The triple scan technique for assessing the internal gap of the 3D-printed prosthesis: (a) image matching of scans, (b) measurement plane formation, and (c) cross-sectional image in the abutment area.

The geometric accuracy of 3D-printed prostheses was evaluated using a vertical marginal gap, internal gap, and intermolar distance. For the marginal gap assessment, the cured prosthesis was passively fitted on the stone model, and the model with prosthesis was positioned perpendicular to the table of the stereomicroscope (EGVM-452M; EG Tech, Seoul, Korea) using utility wax. The midbuccal and lingual margin areas in all abutments were then imaged three times at a magnification of 60x with the stereomicroscope, and each value was determined by averaging three measurements. The measurement value was defined as the vertical marginal discrepancy that was vertical marginal misfit measured parallel to the path of draw of the prosthesis [19]. For the internal gap assessment, a triple-scan technique was used, with three digital scans taken using a structured light scanner (Breuckmann SmartScan; AICON 3D Systems GmbH, Braunschweig, Germany) [5]. The first scan was of the cured prosthesis alone, the second was of the study model, and the third was of the prosthesis on the study model. The data of the three scans were delivered to an image analysis software program (Geomagic DesignX; 3D Systems, Rock Hill, SC, USA), where the three scan images were merged using an area-designated best-fit image matching (Figure 5(a)) [20]. The image alignment to the closest fit with the corresponding images was enabled using an iterative closest point (ICP) algorithm [21]. The cross-sectional line images were buccolingually obtained at the midpoint of the abutment (Figure 5(b)), and the internal gap, the perpendicular distances from the external surface of the abutment to the internal surface of the prosthesis, was measured at the center points of the buccal, lingual, and occlusal aspects (Figure 5(c)). For the intermolar distance assessment, a virtual cross-sectional plane passing through two central fossae of the first molars on both sides was created, and the distance between the most external points of the buccal surfaces of the left and right first molars was measured and compared with that of the prosthesis design image using the image analysis software program. All measurements for evaluating the accuracy of 3D-printed prostheses were carried out by a single examiner blinded to the research objective.

### 2.4. Statistical Analysis

All outcome variable data were reported as mean ± standarddeviation. The Kruskal-Wallis test was used for the comparison of the results among groups that used different postcuring methods using the IBM Statistical Package for the Social Sciences (SPSS) v25.0 for Windows (IBM Corp., Chicago, IL, USA). The statistical significance level was set at 0.05. The Mann-Whitney *U* test with Bonferroni correction was used for post hoc analyses (*α* = 0.017).

## 3. Results

**Figure 6:**
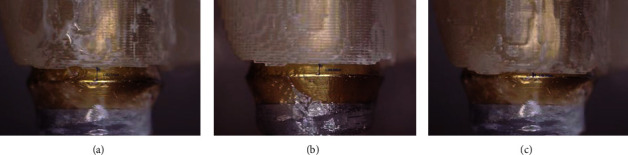
Microscopic image for marginal gap measurement: (a) prosthesis alone, (b) prosthesis with supports, and (c) prosthesis on the stone model.

**Figure 7:**
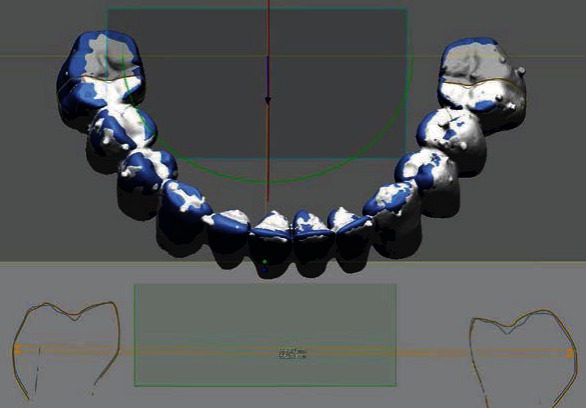
Evaluation of discrepancy of the intermolar distance between 3D-printed and design image of prosthesis.

Table 2 presents the results of fit and dimensional discrepancy of the 3D-printed acrylic prostheses at each measurement point. In general, the PM group showed the lowest mean discrepancy, followed by the PS and P groups. The PS and P groups showed no significant difference in any measurement outcome. The highest discrepancy was found in the measurements of the occlusal area, especially in the P group.  Figure 6 shows images of the marginal gap in the different postcuring groups. The PM group exhibited significantly smaller marginal gaps than the other groups.  Figure 7 shows the outlines of the molars of 3D-printed prostheses and the design image in the cross-sectional view. Again, the discrepancy of intermolar distance was the smallest in the PM group.

## 4. Discussion

This study was designed to find a postcuring method that minimizes the adverse dimensional change for 3D-printed polymeric prostheses. The results showed that the adaptation and dimension of cured prostheses were most accurate when the printed prostheses were cured with seating on the stone model. Thus, the null hypothesis that the differences in the postcuring methods for printed polymeric prostheses would not affect the accuracy of their fabrication was rejected. The mean marginal gap of prostheses in the PM group was lower than 120 *μ*m, which was in a clinically acceptable range. The error in the intermolar distance was significantly smaller in the PM group than the other groups of postcuring without the model. Leaving the supports did not significantly decrease the dimensional error that happened in the postcuring process.

During the printing process, each layer is briefly exposed to curing light and partially solidified. This incomplete polymerization is needed to allow fusion between layers [22]. After the printing process, postcuring is performed to achieve the maximum strength and full density of the material [23]. Chemically, the polymerization of resin material is the increase of the conversion degree, which is a chemical structure change from carbon double bonds (C=C) to carbon single (C-C) bonds [24]. The change in chemical structure inevitably involves a dimensional change of the object [23, 25]. Accordingly, the amount of deformation in postcuring could be affected by the change in the conversion degree. The findings of the present study showed that deformation during postcuring could be minimized when the 3D-printed prosthesis was seated on the abutments of the stone model, leading to markedly low misfit and dimensional error. This might happen because the underlying abutments played the role of mechanical guides that blocked unwanted deformation. Therefore, it is recommended that 3D-printed prostheses are placed on the abutments of the model during the postcuring process. This method may help fabricate prostheses that are closely fitted to abutments in the mouth, in addition to less cement leakage, less need for occlusal adjustment, and better performance of the prosthesis in the long term [26].

The novelty of this study is that it is the first to investigate the impact of postcuring methods on dimensional accuracy in the 3D printing of full-arch polymeric fixed prostheses. Although the design of this study was controlled, there are several limitations derived from its *in vitro* nature. Comprehensive clinical studies including tooth-supported and implant-supported conditions are necessary, and the clinical marginal and internal fit of prostheses needs to be assessed to confirm the findings of the present study. In addition, the deformation phenomenon that occurs during the postcuring process should be evaluated using different 3D printing methods, such as digital light processing, fused deposition modeling, and polymer jetting. Photopolymers are composed of oligomers, monomers, and photoinitiators, and the curing of photopolymers is affected by wavelength, power of light, and radiation time. Thus, further studies on materials and curing setting are needed to optimize the postcuring process.

## 5. Conclusions

Within the limitations of this study, the postcuring process affects the fit and dimensional accuracy of 3D-printed polymeric prostheses. Seating of the prosthesis on the stone model is recommended to minimize the deformity of the prosthesis during the postcuring process.

## Figures and Tables

**Table 1:** Composition of photopolymer used^∗^.

Component	CAS No.	%
*α*,*α*′-[(1-Methylethylidene)di-4,1-phenylene]bis[*ω*-[(2-methyl-1-oxo-2-propenyl)oxy]poly(oxy-1,2-ethanediyl)	41637-38-1	20~35
7,7,9(or 7,9,9)-Trimethyl-4,13-dioxo-3,14-dioxa-5,12-diazahexadecane-1,16-diyl 2-methyl-2-propenoate	72869-86-4	20~28
2-Methyl-2-propenoic acid 1,2-ethanediylbis(oxy-2,1-ethanediyl) ester	109-16-0	20~25
Phenylbis(2,4,6-trimethylbenzoyl)phosphine oxide	162881-26-7	1~10
Rutile (TiO_2_)	1317-80-2	0.1 ~ 5

^∗^Manufacturer's information.

**Table 2:** Discrepancy values (mean ± standarddeviation; *μ*m) of 3D-printed polymeric fixed prostheses fabricated by different postcuring methods.

Area	Postcuring methods			*P*
	Prosthesis alone	Prosthesis with supports	Prosthesis on the stone model	
Margin, buccal	274.4 ± 64.4^a^	233.0 ± 40.3^a^	91.8 ± 27.4^b^	0.008
Axial, buccal	122.1 ± 51.6	125.4 ± 47.8	94.7 ± 64.5	0.468
Occlusal	332.8 ± 70.7^a^	311.7 ± 58.2^a^	126.3 ± 27.3^b^	0.009
Axial, lingual	138.6 ± 46.7^a^	134.3 ± 28.4^a^	64.4 ± 22.2^b^	0.019
Margin, lingual	256.4 ± 46.2^a^	196.8 ± 38.7^a^	89.0 ± 26.7^b^	0.004
Intermolar	115.4 ± 25.3^a^	105.9 ± 12.9^a^	39.2 ± 17.7^b^	0.008

Different superscript lowercase letters indicate significant differences within a row (*α* = 0.05).

## Data Availability

The data used to support the findings of this study are included within the article.
